# 4-{2-Meth­oxy-6-[(4-methyl­phen­yl)imino­meth­yl]phen­oxy}phthalonitrile

**DOI:** 10.1107/S1600536809015402

**Published:** 2009-04-30

**Authors:** Serap Yazıcı, Abdullah Akkaya, Erbil Ağar, İsmet Şenel, Orhan Büyükgüngör

**Affiliations:** a Department of Physics, Faculty of Arts and Sciences, Ondokuz Mayıs University, TR-55139 Kurupelit–Samsun, Turkey; b Department of Chemistry, Faculty of Arts and Sciences, Ondokuz Mayıs University, TR-55139 Kurupelit–Samsun, Turkey

## Abstract

In the mol­ecule of the title compound, C_23_H_17_N_3_O_2_, the methoxy­phenyl ring is oriented at dihedral angles of 13.34 (12) and 88.83 (12)° with respect to the methyl­phenyl and phthalonitrile rings, respectively; the dihedral angle between methyl­phenyl and phthalonitrile rings is 89.67 (10)°. In the crystal structure, weak inter­molecular C—H⋯N inter­actions link mol­ecules into chains. A weak C—H⋯π inter­action is also found..

## Related literature

For a related structure, see: Ocak İskeleli *et al.* (2005 ▶). For general background to substituted phthalonitriles, see: McKeown (1998 ▶); Leznoff & Lever (1989–1996 ▶). For bond-length data, see: Allen *et al.* (1987 ▶).
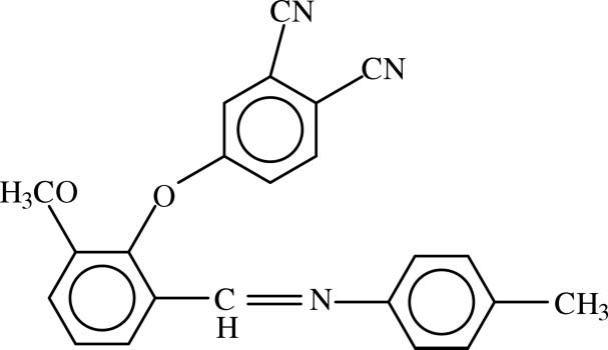


## Experimental

### 

#### Crystal data

C_23_H_17_N_3_O_2_
*M* *_r_* = 367.40
Monoclinic, 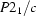

*a* = 9.3549 (5) Å
*b* = 23.6606 (13) Å
*c* = 8.9317 (5) Å
β = 97.256 (4)°
*V* = 1961.13 (19) Å^3^
*Z* = 4
Mo *K*α radiation
μ = 0.08 mm^−1^
*T* = 296 K
0.67 × 0.36 × 0.20 mm

#### Data collection

Stoe IPDS-II diffractometer
Absorption correction: integration (*X-RED32*; Stoe & Cie, 2002 ▶) *T*_min_ = 0.703, *T*_max_ = 0.952
10306 measured reflections
3680 independent reflections
1962 reflections with *I* > 2σ(*I*)
*R*_int_ = 0.072

#### Refinement

*R*[*F* ^2^ > 2σ(*F* ^2^)] = 0.048
*wR*(*F* ^2^) = 0.122
*S* = 0.96
3680 reflections
253 parameters
H-atom parameters constrained
Δρ_max_ = 0.12 e Å^−3^
Δρ_min_ = −0.10 e Å^−3^

### 

Data collection: *X-AREA* (Stoe & Cie, 2002 ▶); cell refinement: *X-AREA*; data reduction: *X-RED32* (Stoe & Cie, 2002 ▶); program(s) used to solve structure: *SHELXS97* (Sheldrick, 2008 ▶); program(s) used to refine structure: *SHELXL97* (Sheldrick, 2008 ▶); molecular graphics: *ORTEP-3 for Windows* (Farrugia, 1997 ▶) and *PLATON* (Spek, 2009 ▶); software used to prepare material for publication: *WinGX* (Farrugia, 1999 ▶).

## Supplementary Material

Crystal structure: contains datablocks I, global. DOI: 10.1107/S1600536809015402/hk2671sup1.cif

Structure factors: contains datablocks I. DOI: 10.1107/S1600536809015402/hk2671Isup2.hkl

Additional supplementary materials:  crystallographic information; 3D view; checkCIF report

## Figures and Tables

**Table 1:** Hydrogen-bond geometry (Å, °)

*D*—H⋯*A*	*D*—H	H⋯*A*	*D*⋯*A*	*D*—H⋯*A*
C4—H4⋯N2^i^	0.93	2.62	3.483 (3)	154
C18—H18⋯*Cg*2^ii^	0.93	2.77	3.694 (3)	171

Symmetry codes: (i) 
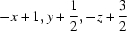
; (ii) 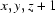. *Cg*2 is the centroid of the C9–C14 ring.

**Fig. 1.**
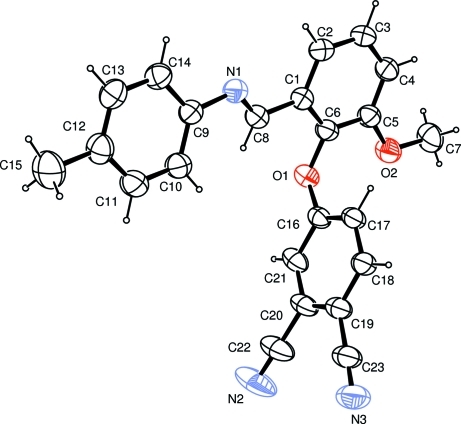
The molecular structure of the title molecule, with the atom-numbering scheme. Displacement ellipsoids are drawn at the 30% probability level.

**Fig. 2.**
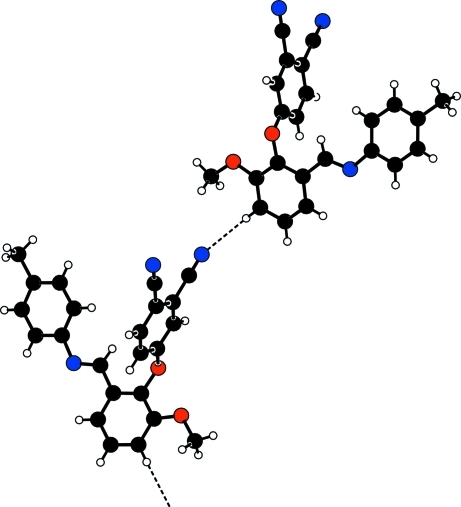
A partial packing diagram of the title compound. Hydrogen bonds are shown as dashed lines.

## supplementary crystallographic information

### Comment

Substituted phthalonitriles are generally used for preparing symmetrically and unsymmetrically peripherally and non-peripherally substituted phthalocyanines and subphthalocyanines (McKeown, 1998; Leznoff & Lever, 1989-1996). In addition to their extensive use as dyes and pigments, phthalocyanines have found widespread applications in catalysis, in optical recording, as photoconductive materials, in photo-dynamic therapy and as chemical sensors (Leznoff & Lever, 1989-1996). We report herein the crystal structure of the title compound.

In the molecule of the title compound (Fig. 1), the bond lengths (Allen *et al.*,  1987) and angles are within normal ranges. The N2≡C22 [1.133 (3) Å] and N3≡C23 [1.145 (3) Å] bonds show N≡C triple bond character and are in good agreement with the literature values (Ocak İskeleli *et al.*, 2005). Rings A (C1-C6), B (C9-C14) and C (C16-C21) are, of course, planar, and they are oriented at dihedral angles of A/B = 13.34 (12), A/C = 88.83 (12) and B/C = 89.67 (10) °.

In the crystal structure, weak intermolecular C-H···N interactions (Table 1) link the molecules into chains (Fig. 2), in which they may be effective in the stabilization of the structure. There also exists a weak C-H···π interaction (Table 1).

### Experimental

For the preparation of the title compound, potasium carbonato (0.9 g, 6.58 mmol) was added to a solution of solid *o*-vaniline (0.5 g, 3.29 mmol) in DMF. The mixture was stirred for 30 min under nitrogen atmosphere. 4-Nitrophtalonitrile solution in DMF was added. The mixture was stirred for 48 h at 323 K under nitrogen atmosphere and poured into ice-water (150 g). The product 2-(3,4-dicyanophenoxy)-3-methoxybenzaldehyde was filtered off and washed with water. The title compound was prepared by refluxing a mixture of a solution containing 2-(3,4-Dicyanophenoxy)-3-methoxybenzaldehyde (0.5 g, 1.799 mmol) in ethanol (20 ml) and a solution containing 4-methylaniline (0.218 g 1.799 mmol) in ethanol (20 ml). The reaction mixture was stirred for 1 h under reflux. Crystals suitable for X-ray analysis were obtained from ethylalcohol by slow evaporation (yield; 55%, m.p. 427-429 K).

### Refinement

H atoms were positioned geometrically, with C-H = 0.93 and 0.96 Å for aromatic and methyl H, respectively, and constrained to ride on their parent atoms, with U_iso_(H) = xU_eq_(C), where x = 1.5 for methyl H and x = 1.2 for aromatic H atoms.

### Crystal data

**Table:**

C_23_H_17_N_3_O_2_	*F*(000) = 768
*M**_r_* = 367.40	*D*_x_ = 1.244 Mg m^−^^3^
Monoclinic, *P*2_1_/*c*	Mo *K*α radiation, λ = 0.71073 Å
Hall symbol: -P 2ybc	Cell parameters from 9188 reflections
*a* = 9.3549 (5) Å	θ = 1.7–26.2°
*b* = 23.6606 (13) Å	µ = 0.08 mm^−^^1^
*c* = 8.9317 (5) Å	*T* = 296 K
β = 97.256 (4)°	Prism, yellow
*V* = 1961.13 (19) Å^3^	0.67 × 0.36 × 0.20 mm
*Z* = 4	

### Data collection

**Table:**

Stoe IPDS-II diffractometer	3680 independent reflections
Radiation source: fine-focus sealed tube	1962 reflections with *I* > 2σ(*I*)
graphite	*R*_int_ = 0.072
Detector resolution: 6.67 pixels mm^-1^	θ_max_ = 25.6°, θ_min_ = 1.7°
ω scans	*h* = −11→10
Absorption correction: integration (*X-RED32*; Stoe & Cie, 2002)	*k* = −28→28
*T*_min_ = 0.703, *T*_max_ = 0.952	*l* = −10→10
10306 measured reflections	

### Refinement

**Table:**

Refinement on *F*^2^	Primary atom site location: structure-invariant direct methods
Least-squares matrix: full	Secondary atom site location: difference Fourier map
*R*[*F*^2^ > 2σ(*F*^2^)] = 0.048	Hydrogen site location: inferred from neighbouring sites
*wR*(*F*^2^) = 0.122	H-atom parameters constrained
*S* = 0.96	*w* = 1/[σ^2^(*F*_o_^2^) + (0.0491*P*)^2^] where *P* = (*F*_o_^2^ + 2*F*_c_^2^)/3
3680 reflections	(Δ/σ)_max_ < 0.001
253 parameters	Δρ_max_ = 0.12 e Å^−^^3^
0 restraints	Δρ_min_ = −0.10 e Å^−^^3^

### Special details

**Table:**

Experimental. 140 frames, detector distance = 130 mm
Geometry. All e.s.d.'s (except the e.s.d. in the dihedral angle between two l.s. planes) are estimated using the full covariance matrix. The cell e.s.d.'s are taken into account individually in the estimation of e.s.d.'s in distances, angles and torsion angles; correlations between e.s.d.'s in cell parameters are only used when they are defined by crystal symmetry. An approximate (isotropic) treatment of cell e.s.d.'s is used for estimating e.s.d.'s involving l.s. planes.
Refinement. Refinement of *F*^2^ against ALL reflections. The weighted *R*-factor *wR* and goodness of fit *S* are based on *F*^2^, conventional *R*-factors *R* are based on *F*, with *F* set to zero for negative *F*^2^. The threshold expression of *F*^2^ > σ(*F*^2^) is used only for calculating *R*-factors(gt) *etc*. and is not relevant to the choice of reflections for refinement. *R*-factors based on *F*^2^ are statistically about twice as large as those based on *F*, and *R*- factors based on ALL data will be even larger.

### Fractional atomic coordinates and isotropic or equivalent isotropic displacement parameters (Å^2^)

**Table:**

	*x*	*y*	*z*	*U*_iso_*/*U*_eq_	
O1	0.38836 (16)	0.44741 (6)	0.57255 (15)	0.0725 (4)	
O2	0.49525 (18)	0.52245 (6)	0.77461 (17)	0.0879 (5)	
N1	0.0395 (2)	0.47793 (7)	0.26224 (19)	0.0737 (5)	
N2	0.4483 (3)	0.21068 (11)	0.7035 (4)	0.1657 (14)	
N3	0.1213 (3)	0.23410 (10)	0.9455 (3)	0.1180 (9)	
C1	0.2188 (2)	0.51464 (8)	0.4521 (2)	0.0659 (6)	
C2	0.1712 (3)	0.57046 (9)	0.4401 (2)	0.0768 (6)	
H2	0.0969	0.5805	0.3657	0.092*	
C3	0.2339 (3)	0.61054 (9)	0.5379 (2)	0.0798 (7)	
H3	0.2028	0.6478	0.5273	0.096*	
C4	0.3424 (3)	0.59675 (9)	0.6520 (2)	0.0760 (7)	
H4	0.3828	0.6245	0.7181	0.091*	
C5	0.3905 (2)	0.54177 (9)	0.6673 (2)	0.0687 (6)	
C6	0.3289 (2)	0.50179 (8)	0.5654 (2)	0.0644 (5)	
C7	0.5596 (3)	0.56217 (11)	0.8831 (3)	0.1040 (9)	
H7A	0.6256	0.5429	0.9569	0.156*	
H7B	0.4859	0.5800	0.9320	0.156*	
H7C	0.6106	0.5903	0.8333	0.156*	
C8	0.1525 (3)	0.46997 (9)	0.3529 (2)	0.0727 (6)	
H8	0.1954	0.4344	0.3568	0.087*	
C9	−0.0244 (2)	0.43225 (9)	0.1744 (2)	0.0683 (6)	
C10	−0.0023 (3)	0.37542 (10)	0.2086 (2)	0.0818 (7)	
H10	0.0624	0.3649	0.2917	0.098*	
C11	−0.0759 (3)	0.33451 (10)	0.1202 (3)	0.0872 (7)	
H11	−0.0599	0.2967	0.1450	0.105*	
C12	−0.1731 (3)	0.34819 (11)	−0.0046 (3)	0.0870 (7)	
C13	−0.1944 (3)	0.40459 (11)	−0.0376 (3)	0.0854 (7)	
H13	−0.2589	0.4149	−0.1209	0.102*	
C14	−0.1221 (2)	0.44613 (10)	0.0501 (2)	0.0761 (6)	
H14	−0.1391	0.4839	0.0254	0.091*	
C15	−0.2535 (4)	0.30258 (13)	−0.0997 (4)	0.1312 (12)	
H15A	−0.2834	0.3170	−0.1993	0.197*	
H15B	−0.3367	0.2913	−0.0543	0.197*	
H15C	−0.1914	0.2706	−0.1059	0.197*	
C16	0.3328 (2)	0.40673 (8)	0.6576 (2)	0.0626 (6)	
C17	0.2221 (3)	0.41613 (8)	0.7412 (2)	0.0674 (6)	
H17	0.1823	0.4520	0.7452	0.081*	
C18	0.1699 (3)	0.37193 (9)	0.8193 (2)	0.0728 (6)	
H18	0.0952	0.3784	0.8767	0.087*	
C19	0.2270 (3)	0.31831 (9)	0.8135 (3)	0.0745 (6)	
C20	0.3403 (3)	0.30971 (9)	0.7282 (3)	0.0793 (7)	
C21	0.3937 (3)	0.35393 (9)	0.6509 (3)	0.0776 (7)	
H21	0.4699	0.3481	0.5951	0.093*	
C22	0.4004 (3)	0.25418 (11)	0.7156 (4)	0.1104 (10)	
C23	0.1688 (3)	0.27176 (10)	0.8882 (3)	0.0904 (8)	

### Atomic displacement parameters (Å^2^)

**Table:**

	*U*^11^	*U*^22^	*U*^33^	*U*^12^	*U*^13^	*U*^23^
O1	0.0718 (10)	0.0594 (9)	0.0864 (10)	0.0058 (8)	0.0110 (8)	−0.0032 (7)
O2	0.0908 (12)	0.0751 (10)	0.0908 (10)	−0.0005 (9)	−0.0153 (9)	−0.0078 (8)
N1	0.0850 (14)	0.0644 (11)	0.0691 (10)	−0.0018 (10)	−0.0001 (10)	0.0018 (9)
N2	0.156 (3)	0.0656 (15)	0.262 (4)	0.0429 (17)	−0.026 (3)	−0.0140 (19)
N3	0.159 (3)	0.0703 (14)	0.1152 (17)	−0.0202 (15)	−0.0180 (16)	0.0176 (12)
C1	0.0758 (15)	0.0581 (12)	0.0637 (12)	−0.0014 (11)	0.0079 (11)	−0.0006 (9)
C2	0.0941 (18)	0.0625 (13)	0.0720 (13)	0.0039 (12)	0.0039 (12)	0.0086 (11)
C3	0.107 (2)	0.0508 (12)	0.0819 (15)	0.0015 (12)	0.0138 (14)	0.0061 (11)
C4	0.0931 (19)	0.0586 (13)	0.0759 (14)	−0.0114 (13)	0.0091 (13)	−0.0052 (10)
C5	0.0731 (15)	0.0603 (13)	0.0718 (13)	−0.0069 (11)	0.0056 (11)	−0.0004 (10)
C6	0.0700 (14)	0.0518 (11)	0.0722 (13)	0.0001 (11)	0.0119 (11)	0.0013 (10)
C7	0.101 (2)	0.1008 (19)	0.1020 (17)	−0.0055 (16)	−0.0182 (16)	−0.0239 (15)
C8	0.0841 (17)	0.0602 (13)	0.0723 (13)	0.0028 (12)	0.0044 (13)	−0.0034 (10)
C9	0.0737 (15)	0.0658 (13)	0.0644 (12)	−0.0017 (11)	0.0045 (11)	0.0013 (10)
C10	0.0931 (19)	0.0699 (15)	0.0781 (14)	−0.0045 (14)	−0.0053 (13)	0.0021 (11)
C11	0.098 (2)	0.0698 (15)	0.0914 (16)	−0.0100 (14)	0.0039 (14)	−0.0001 (13)
C12	0.0813 (18)	0.0915 (18)	0.0861 (16)	−0.0141 (14)	0.0018 (14)	−0.0090 (14)
C13	0.0804 (18)	0.0994 (19)	0.0735 (15)	−0.0033 (15)	−0.0012 (13)	0.0018 (13)
C14	0.0740 (16)	0.0793 (15)	0.0734 (13)	0.0026 (13)	0.0026 (12)	0.0045 (11)
C15	0.130 (3)	0.120 (2)	0.134 (2)	−0.032 (2)	−0.022 (2)	−0.0259 (19)
C16	0.0624 (14)	0.0501 (11)	0.0714 (13)	0.0010 (10)	−0.0061 (11)	−0.0064 (10)
C17	0.0770 (16)	0.0477 (11)	0.0750 (13)	0.0066 (11)	−0.0003 (12)	−0.0028 (9)
C18	0.0842 (18)	0.0575 (13)	0.0750 (13)	−0.0002 (12)	0.0029 (12)	0.0007 (10)
C19	0.0826 (17)	0.0507 (13)	0.0829 (14)	−0.0001 (12)	−0.0184 (13)	0.0014 (10)
C20	0.0807 (17)	0.0462 (12)	0.1017 (17)	0.0116 (12)	−0.0253 (15)	−0.0064 (11)
C21	0.0691 (16)	0.0583 (13)	0.1014 (16)	0.0125 (12)	−0.0047 (12)	−0.0136 (12)
C22	0.104 (2)	0.0576 (15)	0.159 (3)	0.0155 (15)	−0.0258 (19)	−0.0029 (15)
C23	0.113 (2)	0.0544 (14)	0.0954 (17)	−0.0048 (14)	−0.0198 (15)	0.0066 (12)

### Geometric parameters (Å, °)

**Table:**

C1—C6	1.384 (3)	C11—H11	0.9300
C1—C2	1.393 (3)	C12—C13	1.376 (3)
C1—C8	1.465 (3)	C12—C15	1.513 (3)
C2—C3	1.370 (3)	C13—C14	1.379 (3)
C2—H2	0.9300	C13—H13	0.9300
C3—C4	1.384 (3)	C14—H14	0.9300
C3—H3	0.9300	C15—H15A	0.9600
C4—C5	1.377 (3)	C15—H15B	0.9600
C4—H4	0.9300	C15—H15C	0.9600
C5—O2	1.361 (2)	C16—O1	1.369 (2)
C5—C6	1.386 (3)	C16—C17	1.369 (3)
C6—O1	1.400 (2)	C16—C21	1.378 (3)
C7—O2	1.427 (3)	C17—C18	1.381 (3)
C7—H7A	0.9600	C17—H17	0.9300
C7—H7B	0.9600	C18—C19	1.380 (3)
C7—H7C	0.9600	C18—H18	0.9300
C8—N1	1.262 (3)	C19—C20	1.397 (3)
C8—H8	0.9300	C19—C23	1.431 (4)
C9—C14	1.385 (3)	C20—C21	1.381 (3)
C9—C10	1.389 (3)	C20—C22	1.439 (3)
C9—N1	1.422 (3)	C21—H21	0.9300
C10—C11	1.378 (3)	C22—N2	1.133 (3)
C10—H10	0.9300	C23—N3	1.145 (3)
C11—C12	1.385 (3)		
			
C16—O1—C6	119.67 (16)	C10—C11—H11	119.1
C5—O2—C7	117.49 (18)	C12—C11—H11	119.1
C8—N1—C9	120.00 (19)	C13—C12—C11	117.5 (2)
C6—C1—C2	117.71 (19)	C13—C12—C15	121.6 (2)
C6—C1—C8	120.19 (19)	C11—C12—C15	121.0 (3)
C2—C1—C8	122.1 (2)	C12—C13—C14	121.5 (2)
C3—C2—C1	120.1 (2)	C12—C13—H13	119.2
C3—C2—H2	120.0	C14—C13—H13	119.2
C1—C2—H2	120.0	C13—C14—C9	120.8 (2)
C2—C3—C4	121.4 (2)	C13—C14—H14	119.6
C2—C3—H3	119.3	C9—C14—H14	119.6
C4—C3—H3	119.3	C12—C15—H15A	109.5
C5—C4—C3	119.7 (2)	C12—C15—H15B	109.5
C5—C4—H4	120.2	H15A—C15—H15B	109.5
C3—C4—H4	120.2	C12—C15—H15C	109.5
O2—C5—C4	125.72 (19)	H15A—C15—H15C	109.5
O2—C5—C6	115.83 (19)	H15B—C15—H15C	109.5
C4—C5—C6	118.5 (2)	O1—C16—C17	123.69 (18)
C1—C6—C5	122.64 (19)	O1—C16—C21	115.2 (2)
C1—C6—O1	119.32 (17)	C17—C16—C21	121.1 (2)
C5—C6—O1	117.88 (19)	C16—C17—C18	119.5 (2)
O2—C7—H7A	109.5	C16—C17—H17	120.2
O2—C7—H7B	109.5	C18—C17—H17	120.2
H7A—C7—H7B	109.5	C19—C18—C17	121.0 (2)
O2—C7—H7C	109.5	C19—C18—H18	119.5
H7A—C7—H7C	109.5	C17—C18—H18	119.5
H7B—C7—H7C	109.5	C18—C19—C20	118.6 (2)
N1—C8—C1	122.4 (2)	C18—C19—C23	121.2 (3)
N1—C8—H8	118.8	C20—C19—C23	120.2 (2)
C1—C8—H8	118.8	C21—C20—C19	120.6 (2)
C14—C9—C10	118.1 (2)	C21—C20—C22	118.9 (3)
C14—C9—N1	116.8 (2)	C19—C20—C22	120.4 (3)
C10—C9—N1	125.02 (19)	C16—C21—C20	119.2 (2)
C11—C10—C9	120.3 (2)	C16—C21—H21	120.4
C11—C10—H10	119.9	C20—C21—H21	120.4
C9—C10—H10	119.9	N2—C22—C20	178.9 (4)
C10—C11—C12	121.8 (2)	N3—C23—C19	178.8 (3)
			
C1—C6—O1—C16	−91.4 (2)	C2—C1—C8—N1	−7.8 (3)
C5—C6—O1—C16	93.0 (2)	C14—C9—C10—C11	0.2 (4)
C17—C16—O1—C6	−1.5 (3)	N1—C9—C10—C11	176.3 (2)
C21—C16—O1—C6	176.75 (18)	C9—C10—C11—C12	0.1 (4)
C4—C5—O2—C7	1.3 (3)	C10—C11—C12—C13	−0.2 (4)
C6—C5—O2—C7	−179.0 (2)	C10—C11—C12—C15	−179.6 (3)
C1—C8—N1—C9	−176.67 (18)	C11—C12—C13—C14	−0.1 (4)
C14—C9—N1—C8	−162.8 (2)	C15—C12—C13—C14	179.4 (3)
C10—C9—N1—C8	21.0 (4)	C12—C13—C14—C9	0.4 (4)
C6—C1—C2—C3	0.5 (3)	C10—C9—C14—C13	−0.5 (3)
C8—C1—C2—C3	178.3 (2)	N1—C9—C14—C13	−176.9 (2)
C1—C2—C3—C4	−1.5 (4)	O1—C16—C17—C18	177.77 (19)
C2—C3—C4—C5	0.9 (4)	C21—C16—C17—C18	−0.4 (3)
C3—C4—C5—O2	−179.5 (2)	C16—C17—C18—C19	−0.5 (3)
C3—C4—C5—C6	0.8 (3)	C17—C18—C19—C20	0.8 (3)
C2—C1—C6—C5	1.2 (3)	C17—C18—C19—C23	−176.9 (2)
C8—C1—C6—C5	−176.7 (2)	C18—C19—C20—C21	−0.2 (3)
C2—C1—C6—O1	−174.27 (19)	C23—C19—C20—C21	177.5 (2)
C8—C1—C6—O1	7.9 (3)	C18—C19—C20—C22	−178.3 (2)
O2—C5—C6—C1	178.4 (2)	C23—C19—C20—C22	−0.6 (3)
C4—C5—C6—C1	−1.8 (3)	O1—C16—C21—C20	−177.32 (19)
O2—C5—C6—O1	−6.1 (3)	C17—C16—C21—C20	1.0 (3)
C4—C5—C6—O1	173.70 (19)	C19—C20—C21—C16	−0.7 (3)
C6—C1—C8—N1	169.9 (2)	C22—C20—C21—C16	177.4 (2)

### Hydrogen-bond geometry (Å, °)

**Table:**

*D*—H···*A*	*D*—H	H···*A*	*D*···*A*	*D*—H···*A*
C4—H4···N2^i^	0.93	2.62	3.483 (3)	154
C18—H18···Cg2^ii^	0.93	2.77	3.694 (3)	171

Symmetry codes: (i) −*x*+1, *y*+1/2, −*z*+3/2; (ii) *x*, *y*, *z*+1.
